# Supplementary material for: Reliable cell cycle commitment in budding yeast is ensured by signal integration
Source: eLife. 2015 Jan 15;4:e03977. doi: 10.7554/eLife.03977 (PMC4378612; doi:10.7554/eLife.03977)
Supplement: Figure 2—source data 1. — DOI: http://dx.doi.org/10.7554/eLife.03977.014 [file elife03977s001.docx]

| Parameters | Biological meaning | Value (min^-1^) |
| --- | --- | --- |
| $a_{1}$ | Transcription rate of CLN3 gene | 0.8 |
| $D_{1}$ | Degradation rate of $mRNA_{cln3}$ | 0.2 (M Tyers et al. 1992) |
| $a_{2}$ | Initial translation rate of Cln3 | 8 |
| $\alpha$ | Exponential growth exponent of cell volume | 0.01 (Cipollina et al. 2005; Di Talia et al. 2007) |
| $D_{2}$ | Degradation rate of $Protein_{Cln3}$ | 0.2 (M Tyers et al. 1992; Cross and Blake 1993; Yaglom et al. 1995) |
| *r* | Initial transcription rate of CLN3 gene | 1/3 |
