# Supplementary material for: Reliable cell cycle commitment in budding yeast is ensured by signal integration
Source: eLife. 2015 Jan 15;4:e03977. doi: 10.7554/eLife.03977 (PMC4378612; doi:10.7554/eLife.03977)
Supplement: Figure 4—source data 1. — (B) The Whi5tot intensities and G1 lengths in different nutrient conditions. DOI: http://dx.doi.org/10.7554/eLife.03977.022 [file elife03977s002.docx]

|  | Growth rate (min^-1^) | | | | *WHI5_tot_* intensity (a.u.) | | | |
| --- | --- | --- | --- | --- | --- | --- | --- | --- |
| Nutrient condition | N | Mean | Std. | CV | N | Mean | Std. | CV |
| 2% Glucose + AA | 34 | 0.0072 | 0.0023 | 0.32 | 183 | 123.95 | 46.06 | 0.37 |
| 2% Sucrose + AA | 30 | 0.0065 | 0.0026 | 0.39 | 220 | 114.23 | 43.39 | 0.38 |
| 2% Fructose + AA | 27 | 0.0063 | 0.0022 | 0.35 | 200 | 123.15 | 53.26 | 0.43 |
| 2% Raffinose + AA | 51 | 0.0055 | 0.0019 | 0.34 | 214 | 120.58 | 41.67 | 0.35 |
| 2% Galactose + AA | 39 | 0.0045 | 0.0023 | 0.52 | 194 | 182.11 | 70.91 | 0.39 |
| 2% Maltose + AA | 63 | 0.0021 | 0.0013 | 0.62 | 216 | 282.51 | 67.29 | 0.24 |
| 2% Glycerol + AA | 70 | 0.0026 | 0.0016 | 0.62 | 231 | 261.07 | 90.56 | 0.35 |
| 2% Ethanol + AA | 60 | 0.0020 | 0.0013 | 0.62 | 208 | 330.19 | 97.29 | 0.29 |
| 2% Glucose + (NH_4_)_2_SO_4_ | 134 | 0.0041 | 0.0025 | 0.61 | 272 | 208.69 | 44.10 | 0.21 |
| 0.01% Glucose + AA | 74 | 0.0018 | 0.0011 | 0.63 | 200 | 274.62 | 54.59 | 0.20 |
| 0.005% Glucose + AA | 56 | 0.0017 | 0.0013 | 0.79 | 146 | 319.60 | 77.80 | 0.24 |

**Figure 4—source data 1.**

**(a)The growth rates and *Whi5_tot_* intensities in different nutrient conditions.**

**(b) The *Whi5_tot_* intensities and G1 lengths in different nutrient conditions.**

|  |  |  | *WHI5_tot_* intensity (a.u.) | | | G1 length (min) | | |
| --- | --- | --- | --- | --- | --- | --- | --- | --- |
| Nutrient condition |  | N | Mean | Std. | CV | Mean | Std. | CV |
| 2% Glucose + AA | mother | 61 | 103.49 | 33.26 | 0.32 | 8.33 | 1.84 | 0.22 |
|  | daughter | 57 | 143.24 | 48.00 | 0.34 | 18.57 | 6.70 | 0.36 |
| 2% Sucrose + AA | mother | 55 | 95.47 | 29.26 | 0.31 | 8.31 | 1.69 | 0.20 |
|  | daughter | 59 | 136.77 | 48.62 | 0.36 | 17.97 | 8.11 | 0.45 |
| 2% Fructose + AA | mother | 70 | 101.01 | 37.20 | 0.37 | 8.15 | 2.23 | 0.27 |
|  | daughter | 59 | 167.06 | 58.39 | 0.35 | 18.57 | 8.36 | 0.45 |
| 2% Raffinose + AA | mother | 80 | 105.00 | 29.05 | 0.28 | 9.87 | 3.08 | 0.31 |
|  | daughter | 67 | 151.19 | 48.12 | 0.32 | 27.16 | 11.12 | 0.41 |
| 2% Galactose + AA | mother | 76 | 169.46 | 62.84 | 0.37 | 12.57 | 5.60 | 0.45 |
|  | daughter | 57 | 244.83 | 62.28 | 0.25 | 43.80 | 22.62 | 0.52 |
| 2% Maltose + AA | mother | 78 | 272.28 | 70.18 | 0.26 | 26.93 | 11.50 | 0.43 |
|  | daughter | 51 | 307.65 | 57.72 | 0.19 | 77.64 | 34.84 | 0.45 |
| 2% Glycerol + AA | mother | 85 | 252.34 | 97.00 | 0.38 | 22.48 | 19.42 | 0.86 |
|  | daughter | 46 | 301.87 | 79.45 | 0.26 | 68.63 | 41.62 | 0.61 |
| 2% Ethanol + AA | mother | 76 | 323.15 | 107.90 | 0.33 | 43.14 | 42.94 | 0.99 |
|  | daughter | 58 | 344.28 | 75.90 | 0.22 | 93.56 | 55.46 | 0.59 |
| 2% Glucose + (NH_4_)_2_SO_4_ | mother | 170 | 199.84 | 45.61 | 0.23 | 23.78 | 19.07 | 0.80 |
|  | daughter | 102 | 223.45 | 37.23 | 0.17 | 52.78 | 31.66 | 0.60 |
| 0.01% Glucose + AA | mother | 77 | 268.65 | 47.61 | 0.18 | 37.79 | 17.28 | 0.46 |
|  | daughter | 39 | 283.75 | 61.32 | 0.22 | 83.43 | 38.38 | 0.46 |
| 0.005% Glucose + AA | mother | 70 | 299.09 | 76.38 | 0.26 | 45.80 | 30.58 | 0.67 |
|  | daughter | 47 | 332.87 | 64.03 | 0.19 | 115.69 | 49.04 | 0.42 |
