# Supplementary material for: Reliable cell cycle commitment in budding yeast is ensured by signal integration
Source: eLife. 2015 Jan 15;4:e03977. doi: 10.7554/eLife.03977 (PMC4378612; doi:10.7554/eLife.03977)
Supplement: Supplementary file 1. — (A) Mathematic model of Whi5 kinetics. (B) ODE model of the Start network. DOI: http://dx.doi.org/10.7554/eLife.03977.029 [file elife03977s003.pdf]

Supplementary file 1

A. *Mathematic model of Whi5 kinetics*

The major role of this mathematic model is to analytically derive the correlation between Cln3 and T_{G1} coupled by Whi5 phosphorylation under more general conditions without the assumption of saturation for any enzyme. In this section we simplified the phosphorylation of Whi5 by Cln3 as a single step process. From numerical simulation of Whi5 kinetics and phase diagram of Whi5 (fig. 1), we found that the major part of G1 is the process for the nuclear Whi5 to drop from $Whi5_{tot}$ to $Whi5_c$. Here $Whi5_c$ is defined as the corresponding value of Whi5 in phase diagram when Cln3 equals to $Cln3_c$, below which the positive feedback loop takes effect and greatly accelerate the Start transition.

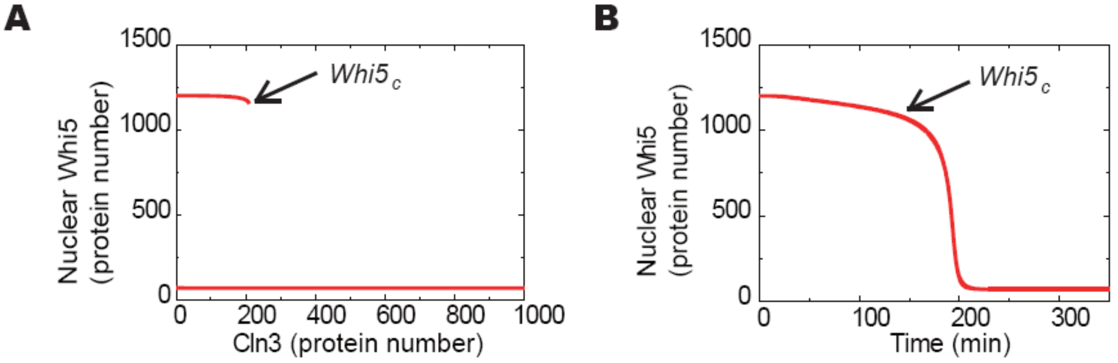

figure 1. (**A**) The phase diagram of Whi5. The red lines show stable state nuclear Whi5 values as a function of Cln3. If the nuclear Whi5 drops below $Whi5_c$, the switch is irreversibly flipped. (**B**) The nuclear Whi5 dynamics at Cln3=200. **A** and **B** are simulated by the ODE model (see section B).

Since the Start transition itself is very quick, we neglected its time in the calculation of T_{G1} for simplicity. We also assume Cln3 concentration is constant through G1. Here we derived the correlation between T_{G1} and Cln3 based on these simplifications. The scheme of the model is shown in fig. 2A, and the equations are listed as follows:

$$\frac{d[Whi5\cdot Cln3]}{dt} = a\cdot[Whi5]\cdot[Cln3]-(k+d)\cdot[Whi5\cdot Cln3]$$

$$\frac{d[Whi5^p]}{dt} = k\cdot[Whi5\cdot Cln3]+f\cdot[Whi5^p\cdot phos]-c\cdot[phos][Whi5^p]$$

$$\frac{d[Whi5^p\cdot phos]}{dt} = c\cdot[Whi5^p]\cdot[phos]-(p+f)\cdot[Whi5^p\cdot phos]$$

Conserved quantity:

$$[Whi5_{tot}] = [Whi5]+[Whi5\cdot Cln3]+[Whi5^p]+[Whi5^p\cdot phos]$$

$$[Cln3_{tot}] = [Cln3] + [Whi5 \cdot Cln3]$$

$$[phos_{tot}] = [phos] + [Whi5^p \cdot phos]$$

Defining $Whi5_{up} \equiv [Whi5] + [Whi5 \cdot Cln3]$ and $Whi5_p \equiv [Whi5^p] + [Whi5^p \cdot phos]$, then $[Whi5_{tot}] = Whi5_{up} + Whi5_p$. We focus on the time interval for $Whi5_{up}$ to drop from $Whi5_{tot}$ to $Whi5_c$.

With Michaelis–Menten approximation

$$\frac{d[Whi5 \cdot Cln3]}{dt} \approx 0, \quad \frac{d[Whi5^p \cdot phos]}{dt} \approx 0,$$

and defining $K_{m1} \equiv \frac{(k+d)}{a}$, $K_{m2} \equiv \frac{(p+f)}{c}$,

we have

$$[Whi5 \cdot Cln3] = \frac{([Cln3_{tot}] + Whi5_{up} + K_{m1})}{2} \cdot \left(1 - \left\{1 - \frac{4 \cdot [Cln3_{tot}] \cdot Whi5_{up}}{([Cln3_{tot}] + Whi5_{up} + K_{m1})^2}\right\}^{1/2}\right)$$

$$\approx \frac{([Cln3_{tot}] + Whi5_{up} + K_{m1})}{2} \cdot \frac{4 \cdot [Cln3_{tot}] \cdot Whi5_{up}}{2 \cdot ([Cln3_{tot}] + Whi5_{up} + K_{m1})^2} = \frac{[Cln3_{tot}] \cdot Whi5_{up}}{([Cln3_{tot}] + Whi5_{up} + K_{m1})}.$$

Similarly, $[Whi5^p \cdot phos] \approx \frac{[phos_{tot}] \cdot Whi5_p}{([phos_{tot}] + Whi5_p + K_{m2})}$.

Thus, $\frac{d[Whi5_p]}{dt} = k \cdot \frac{[Cln3_{tot}] \cdot Whi5_{up}}{([Cln3_{tot}] + Whi5_{up} + K_{m1})} - p \cdot \frac{[phos_{tot}] \cdot Whi5_p}{([phos_{tot}] + Whi5_p + K_{m2})}$.

Since $[Whi5_c] \leq Whi5_{up} \leq [Whi5_{tot}]$ and in our focused range

$$\frac{Whi5_{up}}{([Cln3_{tot}] + Whi5_{up} + K_{m1})} \approx \frac{[Whi5_{tot}] - Whi5_p}{([Cln3_{tot}] + [Whi5_{tot}] + K_{m1})},$$

$$\frac{Whi5_p}{([phos_{tot}] + Whi5_p + K_{m2})} \approx \frac{Whi5_p}{([phos_{tot}] + K_{m2})},$$

the time evolution, of $Whi5_p$ can be solved as:

$$Whi5_p(t) = \int_0^t \left(k \cdot \frac{[Cln3_{tot}] \cdot Whi5_{tot}}{([Cln3_{tot}] + Whi5_{tot} + K_{m1})}\right) \cdot \exp\left(-\int_{t'}^t \left(k \cdot \frac{[Cln3_{tot}]}{([Cln3_{tot}] + Whi5_{tot} + K_{m1})} + \frac{p \cdot [phos_{tot}]}{([phos_{tot}] + K_{m2})}\right) dt''\right) dt'$$

$$Whi5_p(t) = k \cdot \frac{[Cln3_{tot}]\cdot[Whi5_{tot}]}{([Cln3_{tot}]+[Whi5_{tot}]+K_{m1})} \cdot \frac{\left[1-\exp\left(-\left(\frac{p\cdot[phos_{tot}]}{([phos_{tot}]+K_{m2})}+\frac{[Cln3_{tot}]\cdot k}{([Cln3_{tot}]+[Whi5_{tot}]+K_{m1})}\right)\cdot t\right)\right]}{\left\{\frac{p\cdot[phos_{tot}]}{([phos_{tot}]+K_{m2})}+\frac{[Cln3_{tot}]\cdot k}{([Cln3_{tot}]+[Whi5_{tot}]+K_{m1})}\right\}}.$$

Time zero is defined at T_0 in this model.

While by definition $Whi5_p(T_{G1}) = [Whi5_{tot}] - [Whi5_c]$,

we could first get the solution of $Cln3_c$, for $t \to \infty$

$$[Whi5_{tot}] - [Whi5_c] = \frac{k\cdot[Cln3_c]\cdot[Whi5_{tot}]}{\left\{\frac{p\cdot[phos_{tot}]}{([phos_{tot}]+K_{m2})}\cdot([Cln3_c]+[Whi5_{tot}]+K_{m1})+k\cdot[Cln3_c]\right\}}.$$

Thus,

$$[Cln3_c] = ([Whi5_{tot}]+K_{m1})\cdot\left(\frac{1}{([Whi5_{tot}]/[Whi5_c]-1)}\cdot\frac{([phos_{tot}]+K_{m2})\cdot k}{p\cdot[phos_{tot}]}-1\right)^{-1} \quad (1)$$

Considering $[Cln3_{tot}] > [Cln3_c]$, since exp(-x)≈1-x (for x<<1, and can be approximately valid when x<0.5),

for $x = \left(\frac{p\cdot[phos_{tot}]}{([phos_{tot}]+K_{m2})}+\frac{[Cln3_{tot}]\cdot k}{([Cln3_{tot}]+[Whi5_{tot}]+K_{m1})}\right)\cdot t$,

when $T_{G1} < \frac{1}{p}$ and $T_{G1} < ([Cln3_{tot}]+[Whi5_{tot}]+K_{m1})/[Cln3_{tot}]\cdot k$,

by applying exp(-x)≈1-x, we get

$$1-\frac{[Whi5_c]}{[Whi5_{tot}]} \approx k\cdot\frac{[Cln3_{tot}]\cdot T_{G1}}{([Cln3_{tot}]+[Whi5_{tot}]+K_{m1})}.$$

This leads to

$$[Cln3_{tot}]\cdot(T_{G1}-T_c) = A,$$

in which

$$A = \frac{1}{k}\cdot\left(1-\frac{[Whi5_c]}{[Whi5_{tot}]}\right)([Whi5_{tot}]+K_{m1}) \quad (2)$$

and

$$T_c = \frac{1}{k}\cdot\left(1-\frac{[Whi5_c]}{[Whi5_{tot}]}\right) \quad (3)$$

T_c is the minimum G1 length from cytokinesis when Cln3 concentration gets saturated.

Since when $[Cln3_{tot}] \to [Cln3_c]$, $T_{G1} \to +\infty$, we include $[Cln3_c]$ term and put it as

$$([Cln3_{tot}]-[Cln3_c])\cdot(T_{G1}-T_c) = A \quad (4)$$

The value of $Cln3_c$ can be calculated from equation (1). A and T_c are determined from equation (2) and (3), respectively. Thus all parameters in equation (4) are known.

By comparing $Cln3$-T_{G1} curve derived from equation (4) with the deterministic simulation result from the full equations above (with the same set of parameters), we see that the analytical curve coincides with the numerical result (fig. 2B). Similar result can be deduced analytically when Whi5 has multiple phosphorylation sites.

In summary, the inverse correlation between Cln3 concentration and G1 length holds in general under the following condition: $T_{G1} < \min(\frac{m}{k}, \frac{1}{p})$, where k and p are the catalytic rate for Cln3-CDK and the phosphatase, respectively and $m \approx [Whi5]/[Cln3] \approx 10$. For weak kinase and phosphatase, it is quite conceivable that G1 length would satisfy this condition.

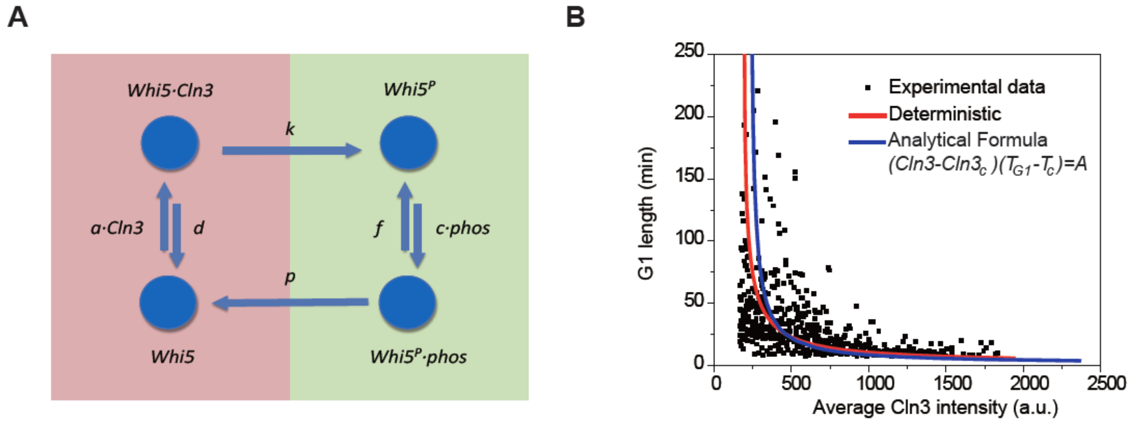

figure 2. (**A**) Single-step phosphorylation of Whi5; *phos* denotes basal phosphatase; *a*, *d*, *c*, *f*, *k* and *p* denote reaction rates. (**B**) Comparison of analytical results with the deterministic simulation of the full equations and the experimental data. Values of $Cln3_c$, T_0 and A in the Analytical Formula are derived from equation (1) to (3).

Predictions of the kinetics model

From equation (1) and equation (2), it is clear that both $Cln3_c$ and A increase monotonously with the increase of $Whi5_{tot}$, while the intensity of dephosphorylation plays important role in elevating $Cln3_c$.

Cln3c changes with whi5tot and positive feedback strength

START transition is triggered when phosphorylated Whi5 level reaches $Whi5_{tot} - Whi5_c$.

From equation (1),

$$Cln3_c = \frac{([Whi5_{tot}]+K_{m1})}{V\cdot k-1},\ V \equiv \frac{Whi5_c}{Whi5_{tot}-Whi5_c}\cdot\frac{([phos_{tot}]+K_{m2})}{p\cdot[phos_{tot}]}.$$

When $Whi5_{tot}$ increases, $Whi5_c$ is almost uninfluenced, thus V drops. The denominator term in equation (1) drops while the numerator term increases, resulting in the elevation of $Cln3_c$.

When positive feedback is weaken by the deletion of *CLN1* or *CLN2*, the value of $Whi5_c$ drops (fig. 3), thus V drops, leading to the elevation of $Cln3_c$.

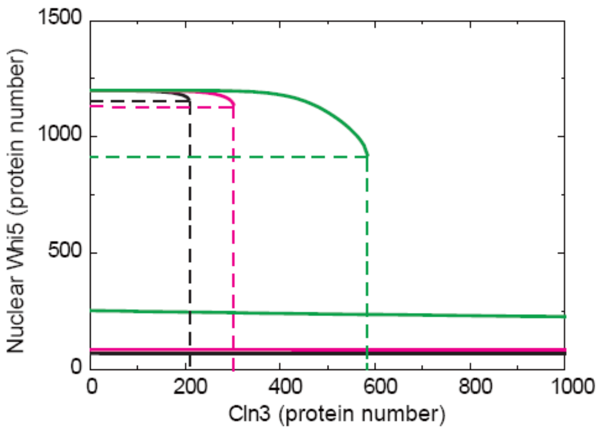

figure 3. $Whi5_c$ and $Cln3_c$ in *cln1Δ* (pink) and *cln2Δ* (green) strains in comparison with strain of endogenous positive feedback loop (black). Nuclear Whi5 stable states at different Cln3 protein numbers (solid lines) were simulated by the ODE model (see Section B). $Whi5_c$ and $Cln3_c$ are indicated by horizontal and vertical dashed lines, respectively. $Whi5_{tot}$ is the nuclear Whi5 level when Cln3 level is zero. $Whi5_c^p$ is the difference from $Whi5_{tot}$ to $Whi5_c$.

Effect of phosphatase having multiple substrates

We also considered the situation that Whi5 may share phosphatase with many other substrates:

$$\frac{dWhi5_p}{dt} = k\cdot Cln3 - p\cdot Phos\cdot\frac{Whi5_p}{K+Whi5_p+X+Y+Z+...},$$

Focus range: time interval for $Whi5_p$ rise from 0 to $Whi5_{tot}$ -$Whi5_c$.

It is appropriate to apply first order kinetics approximation for phosphatase:

$$p\cdot Phos\cdot\frac{Whi5_p}{K+Whi5_p+X+Y+Z+...} \approx p\cdot Phos\cdot\frac{Whi5_p}{K'+X+Y+Z+...}$$

where X, Y, Z are other substrates.

$$\frac{dWhi5_p}{dt} = k\cdot Cln3 - p'\cdot Whi5_p,$$

where $p' = p\cdot Phos/(K'+X+Y+Z+...)$.

So $Whi5_p = \frac{Cln3}{k'}(1-\exp(-k'T)) \approx Cln3 \cdot T$,

which means the integration mechanism holds for the time window $T<1/k'$, where k' can be quite small for weak phosphatase and/or with many substrates. Considering the major phosphatase of Whi5, Cdc14 is sequestered in nucleolus by RENT complex in G1 phase (Shou et al. 1999), the dephosphorylation of Whi5 in G1 should be weak and basal. The half dephosphorylation time of Whi5 in G1 is indeed longer than 30 min (Charvin, Oikonomou, and Siggia 2010), supporting our assumption of a weak and slow dephosphorylation. Thus the integration mechanism still holds even if the phosphatase has multiple substrates.

Whi5 dynamics when considering Cln3 flucturation with time

When Cln3 concentration changes with time, the dynamics of Whi5 phosphorylation is as following:

$$\frac{d\left[Whi5_p(t)\right]}{dt} = k \cdot \frac{\left[Cln3(t)\right] \cdot Whi5_{up}(t)}{\left(\left[Cln3(t)\right] + Whi5_{up}(t) + K_{m1}\right)} - p \cdot \frac{\left[phos_{tot}\right] \cdot Whi5_p(t)}{\left(\left[phos_{tot}\right] + Whi5_p(t) + K_{m2}\right)}$$

$$\frac{d\left[Whi5_p(t)\right]}{dt} = Q(t) - P(t) \cdot Whi5_p(t),\ Whi5_p(t=0) = 0$$

$$\Rightarrow Whi5_p(G_1) = \int_0^{G_1} Q(t) \cdot \exp\left(-\int_t^{G1} P(t')dt'\right) dt$$

Focus range: time interval for $Whi5_P$ rise from 0 to $Whi5_{tot}$ -$Whi5_c$.

Condition 1: Zero-th order kinetics approximation for kinase: $\left[Cln3(t)\right] + K_{m1} \ll Whi5_{up}$,

Zero-th order kinetics approximation for phosphatase: $\left[phos_{tot}\right] + K_{m2} \ll Whi5_p(t)$.

$$\frac{d\left[Whi5_p(t)\right]}{dt} \approx k \cdot \left[Cln3(t)\right] - p \cdot \left[phos_{tot}\right]$$

$$\Rightarrow Q(t) = k \cdot \left[Cln3(t)\right] - p \cdot \left[phos_{tot}\right], P(t) = 0$$

$$\Rightarrow Whi5_p(G_1) = \int_0^{G_1} \left(k \cdot \left[Cln3(t)\right] - p \cdot \left[phos_{tot}\right]\right) dt$$

Condition 2: Zero-th order kinetics approximation for kinase: $\left[Cln3(t)\right] + K_{m1} \ll Whi5_{up}$,

First order kinetics approximation for phosphatase: $p \cdot \frac{\left[phos_{tot}\right] \cdot Whi5_p(t)}{\left(\left[phos_{tot}\right] + Whi5_p(t) + K_{m2}\right)}$

$\approx p' \cdot \left[phos_{tot}\right] \cdot Whi5_p(t)$.

$$\frac{d\left[Whi5_p(t)\right]}{dt} \approx k \cdot \left[Cln3(t)\right] - p' \cdot \left[phos_{tot}\right] \cdot Whi5_p(t)$$

$$\Rightarrow Q(t) = k \cdot \left[Cln3(t)\right],\ P(t) = p' \cdot \left[phos_{tot}\right]$$

$$\Rightarrow Whi5_p(G_1)=\int_0^{G_1} k\cdot\left[Cln3(t)\right]\exp\left(-p'\cdot\left[phos_{tot}\right]\cdot(G_1-t)\right)dt$$

Condition 3: First order kinetics approximation for kinase: $k\cdot\frac{\left[Cln3(t)\right]\cdot Whi5_{up}(t)}{\left(\left[Cln3(t)\right]+Whi5_{up}(t)+K_{m1}\right)}$

$\approx k'\cdot\left[Cln3(t)\right]\cdot Whi5_{up}(t)$,

First order kinetics approximation for phosphatase: $p\cdot\frac{\left[phos_{tot}\right]\cdot Whi5_p(t)}{\left(\left[phos_{tot}\right]+Whi5_p(t)+K_{m2}\right)}$

$\approx p'\cdot\left[phos_{tot}\right]\cdot Whi5_p(t)$.

$$\frac{d\left[Whi5_p(t)\right]}{dt}\approx k'\cdot\left[Cln3(t)\right]\cdot\left(Whi5_{tot}-Whi5_p(t)\right)-p'\cdot\left[phos_{tot}\right]\cdot Whi5_p(t)$$

$$\Rightarrow Q(t)=k'\cdot\left[Cln3(t)\right]\cdot Whi5_{tot},\ P(t)=k'\cdot\left[Cln3(t)\right]+p'\cdot\left[phos_{tot}\right]$$

$$\Rightarrow Whi5_p(G_1)=\int_0^{G_1}\left(k'\cdot\left[Cln3(t)\right]\cdot Whi5_{tot}\right)\cdot\exp\left(-\int_t^{G1}\left(k'\cdot\left[Cln3(t')\right]+p'\cdot\left[phos_{tot}\right]\right)dt'\right)dt$$

Condition 4: Large amount of kinase ($Cln3\approx Whi5_{tot}$ or $Cln3\geq Whi5_{tot}$):

$$k\cdot\frac{\left[Cln3(t)\right]\cdot Whi5_{up}(t)}{\left(\left[Cln3(t)\right]+Whi5_{up}(t)+K_{m1}\right)}\approx k\cdot\frac{\left[Cln3(t)\right]\cdot Whi5_{up}(t)}{\left(\left[Cln3(t)\right]+Whi5_{tot}+K_{m1}\right)},$$

First order kinetics approximation for phosphatase: $p\cdot\frac{\left[phos_{tot}\right]\cdot Whi5_p(t)}{\left(\left[phos_{tot}\right]+Whi5_p(t)+K_{m2}\right)}$

$\approx p'\cdot\left[phos_{tot}\right]\cdot Whi5_p(t)$.

$$\frac{d\left[Whi5_p(t)\right]}{dt}=k\cdot\frac{\left[Cln3(t)\right]\cdot\left(Whi5_{tot}-Whi5_p(t)\right)}{\left(\left[Cln3(t)\right]+Whi5_{tot}+K_{m1}\right)}-p'\cdot\left[phos_{tot}\right]\cdot Whi5_p(t)$$

$$\Rightarrow Q(t)=k\cdot\frac{\left[Cln3(t)\right]\cdot Whi5_{tot}}{\left(\left[Cln3(t)\right]+Whi5_{tot}+K_{m1}\right)},\ P(t)=k\cdot\frac{\left[Cln3(t)\right]}{\left(\left[Cln3(t)\right]+Whi5_{tot}+K_{m1}\right)}+p'\cdot\left[phos_{tot}\right]$$

$$\Rightarrow Whi5_p(G_1)=\int_0^{G_1}\left(k\cdot\frac{\left[Cln3(t)\right]\cdot Whi5_{tot}}{\left(\left[Cln3(t)\right]+Whi5_{tot}+K_{m1}\right)}\right)\cdot\exp\left(-\int_t^{G1}\left(k\cdot\frac{\left[Cln3(t')\right]}{\left(\left[Cln3(t')\right]+Whi5_{tot}+K_{m1}\right)}+p'\cdot\left[phos_{tot}\right]\right)dt'\right)dt$$

For wild type yeast, it is very likely that Condition 2 is the case.

Measuring the memory length by Whi5 nuclear entry

$$\frac{dWhi5^p(t)}{dt}=k\cdot Cln3(t)-p\cdot phos\cdot Whi5^p(t),$$

$$\Rightarrow Whi5^{p}(t)=\int_0^t k\cdot Cln3(t')\exp\left(-p\cdot phos\cdot(t-t')\right)dt'$$
.

The memory length is $1/(p\cdot phos)$.

When Cdk1 is inhibited, $\frac{dWhi5(t)}{dt}=p\cdot phos\cdot Whi5^{p}(t)=p\cdot phos\cdot\left(Whi5_{tot}-Whi5(t)\right)$,

$$Whi5(t)=Whi5_{tot}-\left(Whi5_{tot}-Whi5(t=0)\right)\cdot\exp\left(-p\cdot phos\cdot t\right)$$
.

We fitted the dynamics of Whi5 nuclear entry by

$$Whi5(t)=A-B\cdot\exp(-t/\tau)$$
,

where $1/\tau$ is the Whi5 dephosphorylation rate and τ is the memory length. A, B and τ are fitting parameters to get the least square Q

$$Q(A,B,\tau)=\sum_{i=1}^{n}\left(Whi5(t_i)-\left[A-B\cdot\exp(-t_i/\tau)\right]\right)^2$$
.

B. ODE model of the Start network

Elementary reactions:

$$Whi5_{nuc}^{np}+Cln3\underset{d}{\overset{a}{\longleftrightarrow}}Whi5_{nuc}^{np}\cdot Cln3\xrightarrow{k}Whi5_{nuc}^{n+1p}+Cln3\quad n=(0,1,2,3)$$

$$Whi5_{nuc}^{np}+Cln1\underset{e_1}{\overset{b_1}{\longleftrightarrow}}Whi5_{nuc}^{np}\cdot Cln1\xrightarrow{l_1}Whi5_{nuc}^{n+1p}+Cln1\quad n=(0,1,2,3)$$

$$Whi5_{nuc}^{np}+Cln2\underset{e_2}{\overset{b_2}{\longleftrightarrow}}Whi5_{nuc}^{np}\cdot Cln2\xrightarrow{l_2}Whi5_{nuc}^{n+1p}+Cln2\quad n=(0,1,2,3)$$

$$Whi5_{nuc}^{n+1p}+phos_{nuc}\underset{f}{\overset{c}{\longleftrightarrow}}Whi5_{nuc}^{n+1p}\cdot phos_{nuc}\xrightarrow{p}Whi5_{nuc}^{np}+phos_{nuc}\quad n=(0,1,2,3)$$

$$Whi5_{nuc}^{4p}\xrightarrow{ab_1}Whi5_{cyto}^{4p}\xrightarrow[+phos_{cyto}]{ab_2}Whi5_{nuc}^{3p}$$

$$\left[SBF_{active}\right]=\frac{\left[SBF_{tot}\right]}{1+\left(\left[Whi5_{nuc}^{\le 3p}\cdot add\right]/\left(j_3\cdot Whi5_{tot}^{wt}\right)\right)^{n3}}$$

$$Gene_{CLN1}\xrightarrow[+[SBF_{active}]]{v_1}Cln1\xrightarrow{D_1}\therefore$$

$$Gene_{CLN2}\xrightarrow[+[SBF_{active}]]{v_2}Cln2\xrightarrow{D_2}\therefore$$

$$Gene_{CLN3}\xrightarrow[IPTG]{v_3}Cln3\xrightarrow{D_3}\therefore$$

Ordinary Differential Equations:

$$\frac{d\left[Whi5_{nuc}^{n+1p}\right]}{dt}=k\cdot\left[Whi5_{nuc}^{np}\cdot Cln3\right]+l_1\cdot\left[Whi5_{nuc}^{np}\cdot Cln1\right]+l_2\cdot\left[Whi5_{nuc}^{np}\cdot Cln2\right]$$

$$+f\cdot\left[Whi5_{nuc}^{n+1p}\cdot phos_{nuc}\right]+p\cdot\left[Whi5_{nuc}^{n+2p}\cdot phos_{nuc}\right]+d\cdot\left[Whi5_{nuc}^{n+1p}\cdot Cln3\right]$$

$$-\left[Whi5_{nuc}^{n+1p}\right]\cdot\left(c\cdot\left[phos_{nuc}\right]+a\cdot\left[Cln3\right]+b_1\cdot\left[Cln1\right]+b_2\cdot\left[Cln2\right]\right)$$

$$+e_1\cdot\left[Whi5_{nuc}^{n+1p}\cdot Cln1\right]+e_2\cdot\left[Whi5_{nuc}^{n+1p}\cdot Cln2\right]\qquad n=\left(0,1\right)$$

$$\frac{d\left[Whi5_{nuc}^{3p}\right]}{dt}=k\cdot\left[Whi5_{nuc}^{2p}\cdot Cln3\right]+l_1\cdot\left[Whi5_{nuc}^{2p}\cdot Cln1\right]+l_2\cdot\left[Whi5_{nuc}^{2p}\cdot Cln2\right]$$

$$+f\cdot\left[Whi5_{nuc}^{3p}\cdot phos_{nuc}\right]+p\cdot\left[Whi5_{nuc}^{4p}\cdot phos_{nuc}\right]+d\cdot\left[Whi5_{nuc}^{3p}\cdot Cln3\right]$$

$$-\left[Whi5_{nuc}^{3p}\right]\cdot\left(c\cdot\left[phos_{nuc}\right]+a\cdot\left[Cln3\right]+b_1\cdot\left[Cln1\right]+b_2\cdot\left[Cln2\right]\right)+ab_2\left[Whi5_{cyto}^{4p}\right]$$

$$+e_1\cdot\left[Whi5_{nuc}^{3p}\cdot Cln1\right]+e_2\cdot\left[Whi5_{nuc}^{3p}\cdot Cln2\right]$$

$$\frac{d\left[Whi5_{nuc}^{4p}\right]}{dt}=k\cdot\left[Whi5_{nuc}^{3p}\cdot Cln3\right]+l_1\cdot\left[Whi5_{nuc}^{3p}\cdot Cln1\right]+l_2\cdot\left[Whi5_{nuc}^{3p}\cdot Cln2\right]$$

$$+f\cdot\left[Whi5_{nuc}^{4p}\cdot phos_{nuc}\right]-\left[Whi5_{nuc}^{4p}\right]\cdot\left(c\cdot\left[phos_{nuc}\right]+ab_1\right)$$

$$\frac{d\left[Whi5^{np}\cdot Cln3\right]}{dt}=a\cdot\left[Whi5_{nuc}^{np}\right]\cdot\left[Cln3\right]-\left(k+d\right)\cdot\left[Whi5_{nuc}^{np}\cdot Cln3\right]\quad n=\left(0,1,2,3\right)$$

$$\frac{d\left[Whi5^{np}\cdot Cln1\right]}{dt}=b_1\cdot\left[Whi5_{nuc}^{np}\right]\cdot\left[Cln1\right]-\left(e_1+l_1\right)\left[Whi5_{nuc}^{np}\cdot Cln1\right]\quad n=\left(0,1,2,3\right)$$

$$\frac{d\left[Whi5^{np}\cdot Cln2\right]}{dt}=b_2\cdot\left[Whi5_{nuc}^{np}\right]\cdot\left[Cln2\right]-\left(e_2+l_2\right)\left[Whi5_{nuc}^{np}\cdot Cln2\right]\quad n=\left(0,1,2,3\right)$$

$$\frac{d\left[Whi5^{n+1p}\cdot phos_{nuc}\right]}{dt}=c\cdot\left[Whi5_{nuc}^{n+1p}\right]\cdot\left[phos_{nuc}\right]-\left(p+f\right)\cdot\left[Whi5_{nuc}^{n+1p}\cdot phos_{nuc}\right]\quad n=\left(0,1,2,3\right)$$

$$\frac{d\left[Whi5_{cyto}^{4p}\right]}{dt}=ab_1\cdot\left[Whi5_{nuc}^{4p}\right]-ab_2\left[Whi5_{cyto}^{4p}\right]$$

$$\frac{d\left[Cln1_{tot}\right]}{dt}=v_1\frac{\left\{\left[SBF_{active}\right]/\left(j_1\cdot\left[SBF_{tot}\right]\right)\right\}^{n1}}{1+\left\{\left[SBF_{active}\right]/\left(j_1\cdot\left[SBF_{tot}\right]\right)\right\}^{n1}}-D_1\cdot\left[Cln1_{tot}\right]$$

$$\frac{d\left[Cln2_{tot}\right]}{dt}=v_2\frac{\left\{\left[SBF_{active}\right]/\left(j_2\cdot\left[SBF_{tot}\right]\right)\right\}^{n2}}{1+\left\{\left[SBF_{active}\right]/\left(j_2\cdot\left[SBF_{tot}\right]\right)\right\}^{n2}}-D_2\cdot\left[Cln2_{tot}\right]\quad\frac{d\left[Cln3_{tot}\right]}{dt}=v_3-D_3\cdot\left[Cln3_{tot}\right]$$

Conserved quantity:

$$\left[Cln3_{tot}\right]=\left[Cln3\right]+\sum_{i=0}^{3}\left[Whi5_{nuc}^{np}\cdot Cln3\right]$$

$$\left[phos_{nuc}^{tot}\right]=\left[phos_{nuc}\right]+\sum_{n=1}^{4}\left[Whi5_{nuc}^{np}\cdot phos_{nuc}\right]$$

$$\left[Whi5_{tot}\right]=\sum_{n=0}^{3}\left\{\left[Whi5_{nuc}^{np}\right]+\left[Whi5_{nuc}^{np}\cdot Cln3\right]+\left[Whi5_{nuc}^{np}\cdot Cln1\right]+\left[Whi5_{nuc}^{np}\cdot Cln2\right]\right.$$

$$\left.+\left[Whi5_{nuc}^{n+1p}\cdot phos_{nuc}\right]\right\}+\left[Whi5_{cyto}^{4p}\right]+\left[Whi5_{nuc}^{4p}\right]$$

$$\left[SBF_{tot}\right]=\left[SBF_{active}\right]+\left[SBF_{inactive}\right]$$

$$\left[Cln1_{tot}\right]=\left[Cln1\right]+\sum_{i=0}^{3}\left[Whi5_{nuc}^{np}\cdot Cln1\right]$$

$$\left[Cln2_{tot}\right]=\left[Cln2\right]+\sum_{i=0}^{3}\left[Whi5_{nuc}^{np}\cdot Cln2\right]$$

Hypotheses in this model:

1) For *Whi5* multisite phosphorylation, hypotheses A) to D) was assumed:
   A) *Whi5* localization was determined by the phosphorylation state of its 4 key phosphorylation sites (Wagner et al. 2009);
   B) Distributive kinetics in enzyme processivity;
   C) Sequential ordered phosphorylation;
   D) No cooperativity between sites within a molecule.
2) Whi5 degradation was omitted during G1.

Some of the parameters were derived from published data and our own experimental results, as indicated below. Others were found by using Latin Hypercube sampling then changed by hand to improve the fit. The parameters were determined by fitting the *1XWHI5* data in Figure 3A first. Then only T_0 and $Whi5_{tot}$, $Whi5_{tot}$, v_1, v_2, and the dephosphorylation rate ab_2 and p were changed to fit the experimental data for *whi5Δ*, *2XWHI5*, *cln1Δ*, *cln2Δ,* and *CDC14* overexpression in Figure 3A, C and D, respectively. All the parameters are listed in Figure 3—table supplement 1.

Experimental data used for deriving parameters:

1) Copy numbers of proteins in the Start network (Huh et al. 2003).

2) Half-lives of Cln1-3 (Cross and Blake 1993; Wittenberg, Sugimoto, and Reed 1990).

3) T_0 in *whi5Δ* data.

Table. Parameters of the ODE model.

For *1×WHI5* strain

$$a=2.89\times10^{3}\,\mu mol^{-1}\cdot mL\cdot Min^{-1}\qquad b_1=2.19\times10^{4}\,\mu mol^{-1}\cdot mL\cdot Min^{-1}$$

$$b_2=2.19\times10^{4}\,\mu mol^{-1}\cdot mL\cdot Min^{-1}\qquad c=2.19\times10^{4}\,\mu mol^{-1}\cdot mL\cdot Min^{-1}$$

$e_1 = 0.5Min^{-1}$ $e_2 = 0.5Min^{-1}$ $f = 0.3Min^{-1}$ $k = 2Min^{-1}$<br>$l_1 = 10Min^{-1}$ $l_2 = 5Min^{-1}$ $p = 0.34Min^{-1}$ $ab_1 = 1Min^{-1}$<br>$ab_2 = 0.03Min^{-1}$ $D_1 = 0.13Min^{-1}$ $D_2 = 0.13Min^{-1}$ $D_3 = 3.3\times10^{-2}Min^{-1}$<br>$j_{1,2} = 1$ $j_3 = 0.383$ $n_1 = 1$ $n_2 = 1$ $n_3 = 5$<br>$v_1 = 4.5\times10^{-5}\mu mol\cdot mL^{-1}\cdot Min^{-1}$ $v_2 = 1.79\times10^{-4}\mu mol\cdot mL^{-1}\cdot Min^{-1}$<br>$\left[phos_{nuc}^{tot}\right] = 3.43\times10^{-4}\mu mol\cdot mL^{-1}$ $\left[SBF_{tot}\right] = 3.43\times10^{-4}\mu mol\cdot mL^{-1}$<br>$\left[Whi5_{tot}\right] = 6.85\times10^{-4}\mu mol\cdot mL^{-1}$ $Volume_{nuc}^{estimate} = 2.91\mu m^3$
For *whi5Δ* strain<br>$\left[Whi5_{tot}\right] = 0\mu mol\cdot mL^{-1}$ $\left[T_0\right] = 11\text{min}$ added on G1<br>Other parameters are the same as *1×WHI5*
For *2×WHI5* strain<br>$\left[Whi5_{tot}\right] = 1.60\times10^{-3}\mu mol\cdot mL^{-1}$ Other parameters are the same as *1×WHI5*
For *CDC14* overexpressed strain<br>$ab_2 = 0.2Min^{-1}$ $p = 0.45Min^{-1}$ Other parameters are the same as *1×WHI5*
For *cln1Δ* strain<br>$v_1 = 0\mu mol\cdot mL^{-1}\cdot Min^{-1}$ Other parameters are the same as *1×WHI5*
For *cln2Δ* strain<br>$v_2 = 0\mu mol\cdot mL^{-1}\cdot Min^{-1}$ Other parameters are the same as *1×WHI5*

References:

Charvin, Gilles, Catherine Oikonomou, and ED Siggia. 2010. "Origin of Irreversibility of Cell Cycle Start in Budding Yeast." *PLoS Biology* 8 (1) (January): e1000284. doi:10.1371/journal.pbio.1000284.

Cross, Frederick R, and Christa M Blake. 1993. "The Yeast Cln3 Protein Is an Unstable Activator of Cdc28." *Molecular and Cellular Biology* 13 (6): 3266–3271. doi:10.1128/MCB.13.6.3266.

Huh, WK, JV Falvo, LC Gerke, AS Carroll, RW Howson, J S Weissman, and Erin K O'Shea. 2003. "Global Analysis of Protein Localization in Budding Yeast." *Nature* 425 (6959) (October 16): 686–91. doi:10.1038/nature02026.

Shou, W, J H Seol, a Shevchenko, C Baskerville, D Moazed, Z W Chen, J Jang, H Charbonneau, and R J Deshaies. 1999. "Exit from Mitosis Is Triggered by Tem1-Dependent Release of the Protein Phosphatase Cdc14 from Nucleolar RENT Complex." Cell 97 (2) (April 16): 233–44. doi: 10.1016/S0092-8674(00)80733-3.

Wagner, MV, MB Smolka, RAM de Bruin, and Huilin Zhou. 2009. "Whi5 Regulation by Site Specific CDK-Phosphorylation in Saccharomyces cerevisiae." *PLoS One* 4 (1) (January): e4300. doi:10.1371/journal.pone.0004300.

Wittenberg, C, K Sugimoto, and SI Reed. 1990. "G1-Specific Cyclins of S. Cerevisiae: Cell Cycle Periodicity, Regulation by Mating Pheromone, and Association with the p34CDC28 Protein Kinase." *Cell* 62 (2): 225–237. doi: 10.1016/0092-8674(90)90361-H.
