# Supplementary material for: Reliable cell cycle commitment in budding yeast is ensured by signal integration
Source: eLife. 2015 Jan 15;4:e03977. doi: 10.7554/eLife.03977 (PMC4378612; doi:10.7554/eLife.03977)
Supplement: Supplementary file 2. — (A) Yeast Strain list. (B) Plasmid list. DOI: http://dx.doi.org/10.7554/eLife.03977.030 [file elife03977s004.docx]

# Supplementary file 2: Lists of strains and plasmids in this study

#### Yeast strain list

| Name | Genotype | Source or reference |
| --- | --- | --- |
| W303-1A | *MATa leu2-3/112 ura3-1 trp161 his3-11/15 ade2-1 can1-100 GAL SUC2 mal0* | EUROSARF (Jansen et al. 2005) |
| JAU01 | W303-1A *cdc28-as1* | (Bishop et al. 2000) |
| W303-1A ADE2+ | W303-1A *ADE2* | This study |
| YCT2001 | W303-1A ADE2+ *WHI5-tdTomato* | This study |
| YCT2002 | W303-1A ADE2+ *WHI5-tdTomato cln3::NAT-MX6 bck2::KAN-MX6 STE5pr-LAC12-GPDpr-LacI-GlacSpr-CLN3::HIS3* | This study |
| YCT2003 | W303-1A ADE2+ *WHI5-tdTomato cln3::NAT-MX6 bck2::KAN-MX6 STE5pr-LAC12-GPDpr-LacI-GlacSpr-GFP-GFP-CLN3R108A::HIS3* | This study |
| YCT2004 | W303-1A ADE2+ *WHI5-tdTomato cln3::NAT-MX6 bck2::KAN-MX6 STE5pr-LAC12-GPDpr-LacI-GlacSpr-GFP-GFP-CLN3D166A::HIS3* | This study |
| YCT2005 | W303-1A ADE2+ *WHI5-tdTomato cln3::NAT-MX6 bck2::KAN-MX6 STE5pr-LAC12-GPDpr-LacI-GlacSpr-Venus-Venus-Venus-CLN3R108A::HIS3 ADH1pr-HTB2-CFP::URA3* | This study |
| YCT2006 | W303-1A ADE2+ *WHI5-tdTomato ADH1pr-MCM-GFP::URA3* | This study |
| YCT2007 | W303-1A ADE2+ *WHI5-tdTomato cln3::NAT-MX6 bck2::KAN-MX6 STE5pr-LAC12-GPDpr-LacI-GlacSpr-GFP-GFP-CLN3R108A::HIS3 WHI5pr-WHI5-tdTomato::URA3* | This study |
| YCT2008 | W303-1A ADE2+ *cln3::NAT-MX6 bck2::KAN-MX6* *whi5::LEU2 STE5pr-LAC12-GPDpr-LacI-GlacSpr-GFP-GFP-CLN3R108A::HIS3* *ADH1pr-MCM-mCherry::URA3* | This study |
| YCT2009 | W303-1A ADE2+ *WHI5-tdTomato cln3::NAT-MX6 bck2::KAN-MX6 STE5pr-LAC12-GPDpr-LacI-ADHSpr-GFP-GFP-CLN3R108A::HIS3 GPDpr-CDC14::URA3* | This study |
| YCT2010 | W303-1A ADE2+ *cln3::NAT-MX6 bck2::KAN-MX6* *STE5pr-LAC12-GPDpr-LacI-GlacSpr-GFP-GFP-CLN3R108A::HIS3* *ADH1pr-MCM-mCherry::URA3* | This study |
| YCT2011 | W303-1A ADE2+ *cln3::NAT-MX6* | This study |
| YCT2013 | W303-1A ADE2+ *WHI5-tdTomato cln3::NAT-MX6 bck2::KAN-MX6 STE5pr-LAC12-GPDpr-LacI-GlacSpr-GFP-GFP-CLN3R108A::HIS3 cln1::LEU2* | This study |
| YCT2014 | W303-1A ADE2+ *WHI5-tdTomato cln3::NAT-MX6 bck2::KAN-MX6 STE5pr-LAC12-GPDpr-LacI-GlacSpr-GFP-GFP-CLN3R108A::HIS3 cln2::LEU2* | This study |
| YCT2015 | W303-1A ADE2+ *WHI5-tdTomato cln3::NAT-MX6 STE5pr-LAC12-GPDpr-LacI-GlacSpr-GFP-GFP-CLN3R108A::HIS3* | This study |
| YCT2016 | JAU01 *WHI5-tdTomato* | This study |

***B. Plasmid list***

| Name | Description | Source or reference |
| --- | --- | --- |
| pFA6-KAN-MX6 |  | (Longtine et al. 1998) |
| pFA6-NAT-MX6 |  | (Goldstein and McCusker 1999) |
| pRS304 |  | (Sikorski and Hieter 1989) |
| pRS305 |  | (Sikorski and Hieter 1989) |
| pRS306 |  | (Sikorski and Hieter 1989) |
| pGREG506 |  | EUROSARF (<http://web.uni-frankfurt.de/fb15/mikro/euroscarf/>) |
| pNH603 |  | Wendell A. Lim (unpublished) |
| pNT8 |  | Jonathan Weissman (unpublished) |
| pNT10 |  | Jonathan Weissman (unpublished) |
| pVenus-N1-NPY |  | (Kubota, Mikoshiba, and Miyawaki 2002) |
| pKR1B-LAC4-1 |  | (Sreekrishna and Dickson 1985) |
| pRS424-GPD |  | (Mumberg, Müller, and Funk 1995) |
| pRS304-tdToamto |  | Wendell A. Lim (unpublished) |
| pML103 |  | (Liku et al. 2005) |
| [BBa_E0020](http://partsregistry.org/wiki/index.php?title=Part:BBa_E0020) |  | iGEM part |
| pCT2001 | pNI8-WHI5-tdTomato-CaURA3 | This study |
| pCT2002 | pNH603-STE5pr-LAC12-GPDpr-LacI-GlacSpr -CLN3 | This study |
| pCT2003 | pNH603-STE5pr-LAC12-GPDpr-LacI-GlacSpr –GFP-GFP-CLN3R108A | This study |
| pCT2004 | pNH603-STE5pr-LAC12-GPDpr-LacI-GlacSpr –GFP-GFP-CLN3D166A | This study |
| pCT2005 | pNH603-STE5pr-LAC12-GPDpr-LacI-GlacSpr –Venus-Venus-Venus-CLN3R108A | This study |
| pCT2006 | pRS304-ADH1pr-HTB2-CFP | This study |
| pCT2007 | pRS306-ADH1pr-MCM-mCherry | This study |
| pCT2008 | pRS306-ADH1pr-MCM-GFP | This study |
| pCT2009 | pRS306-WHI5pr-WHI5-tdTomato | This study |
| pCT2010 | pRS306-GPDpr-CDC14 | This study |

Reference:

Bishop, AC, JA Ubersax, DT Petsch, DP Matheos, NS Gray, J Blethrow, E Shimizu, et al. 2000. “A Chemical Switch for Inhibitor-Sensitive Alleles of Any Protein Kinase.” *Nature* 407: 395–401. doi :10.1038/35030148.

Goldstein, a L, and J H McCusker. 1999. “Three New Dominant Drug Resistance Cassettes for Gene Disruption in Saccharomyces Cerevisiae.” *Yeast (Chichester, England)* 15 (14) (October): 1541–53. doi:10.1002/(SICI)1097-0061(199910)15:14<1541::AID-YEA476>3.0.CO;2-K.

Jansen, Gregor, Cunle Wu, Babette Schade, David Y Thomas, and Malcolm Whiteway. 2005. “Drag&Drop Cloning in Yeast.” *Gene* 344 (January 3): 43–51. doi:10.1016/j.gene.2004.10.016.

Kubota, Mie, Katsuhiko Mikoshiba, and Atsushi Miyawaki. 2002. “A Variant of Yellow Fluorescent Protein with Fast and Efficient Maturation for Cell-Biological Applications.” *Nature* 20 (1) (January): 87–90. doi:10.1038/nbt0102-87.

Liku, ME, VQ Nguyen, AW Rosales, K Irie, and J J Li. 2005. “CDK Phosphorylation of a Novel NLS-NES Module Distributed between Two Subunits of the Mcm2-7 Complex Prevents Chromosomal Rereplication.” *Molecular Biology of the* 16 (October): 5026–5039. doi:10.1091/mbc.E05.

Longtine, M S, a McKenzie, D J Demarini, N G Shah, a Wach, a Brachat, P Philippsen, and J R Pringle. 1998. “Additional Modules for Versatile and Economical PCR-Based Gene Deletion and Modification in Saccharomyces Cerevisiae.” *Yeast (Chichester, England)* 14 (10) (July): 953–61. doi:10.1002/(SICI)1097-0061(199807)14:10<953::AID-YEA293>3.0.CO;2-U.

Mumberg, D, R Müller, and M Funk. 1995. “Yeast Vectors for the Controlled Expression of Heterologous Proteins in Different Genetic Backgrounds.” *Gene* 156 (1) (April 14): 119–22.

Sikorski, RS, and Philip Hieter. 1989. “A System of Shuttle Vectors and Yeast Host Strains Designed for Efficient Manipulation of DNA in Saccharomyces Cerevisiae.” *Genetics* 122: 19–27.

Sreekrishna, K, and R C Dickson. 1985. “Construction of Strains of Saccharomyces Cerevisiae That Grow on Lactose.” *Proceedings of the National Academy of Sciences of the United States of America* 82 (23) (December): 7909–13.
